# The heterogeneous nuclear ribonucleoprotein (hnRNP) glorund functions in the *Drosophila* fat body to regulate lipid storage and transport

**DOI:** 10.1016/j.bbrep.2021.100919

**Published:** 2021-01-23

**Authors:** Annabella M. Kolasa, Jasleen K. Bhogal, Justin R. DiAngelo

**Affiliations:** Division of Science, Penn State Berks, Reading, PA, USA

**Keywords:** hnRNP, Glo, *Drosophila*, Lipid, Fat body

## Abstract

The availability of excess nutrients in Western diets has led to the overaccumulation of these nutrients as triglycerides, a condition known as obesity. The full complement of genes important for regulating triglyceride storage is not completely understood. Genome-wide RNAi screens in *Drosophila* cells have identified genes involved in mRNA splicing as important lipid storage regulators. Our lab has shown that a group of splicing factors called heterogeneous nuclear ribonucleoproteins (hnRNPs) regulate lipid metabolism in the fly fat body; however, the identities of all the hnRNPs that function to control triglyceride storage are not known. Here, we used the GAL4/UAS system to induce RNAi to the hnRNP *glorund* (*glo*) in the *Drosophila* fat body to assess whether this hnRNP has any metabolic functions. Decreasing *glo* levels resulted in less triglycerides being stored throughout the fly. Interestingly, decreasing fat body *glo* expression resulted in increased triglyceride storage in the fat body, but blunted triglyceride storage in non-fat body tissues, suggesting a defect in lipid transport. Consistent with this hypothesis, the expression of *apolipophorin* (*apolpp*), *microsomal triglyceride transfer protein* (*mtp*), and *apolipoprotein lipid transfer particle* (*apoltp*), apolipoprotein genes important for lipid transport through the fly hemolymph, was decreased in *glo*-RNAi flies, suggesting that *glo* regulates the transport of lipids from the fly fat body to surrounding tissues. Together, these results indicate that *glorund* plays a role in controlling lipid transport and storage and provide additional evidence of the link between gene expression and the regulation of lipid metabolism.

## Highlights

• The metabolic functions of the hnRNP glorund (*glo*) are unknown.
• Fat body *glo*-RNAi increases fat body triglyceride storage.
• Fat body *glo*-RNAi decreases triglyceride storage in non-fat body tissues.
• Decreasing *glo* in the fly fat body blunts the expression of lipid transport genes.

## 1 Introduction

Metabolic diseases like obesity have persisted in affecting people on a global scale, with nearly one-third of the world's population now being classified as either overweight or obese, and an increase in obesity rates for all ages and both sexes [1]. Obesity results from excessive intake of nutrients and overaccumulation of triglycerides in the body. Understanding the mechanisms controlling nutrient intake and the excess storage of triglycerides has become increasingly important as obesity and its comorbidities such as cardiovascular disease and Type II Diabetes increase [2]. A better understanding of the genes and mechanisms regulating the storage of excess triglycerides during nutrient abundance could be impactful in combatting the rise of obesity.

The fruit fly, *Drosophila melanogaster*, has emerged as an excellent model system to study lipid storage and metabolism due to its strong genetic similarity to humans, vast genetic tools available and the ability to store triglycerides in a liver and adipose-like organ called the fat body [3,4]. Many genes that are important for lipid storage have been identified through a series of genome-wide RNAi screens in both cultured *Drosophila* cells and intact flies [[5], [6], [7]]. A number of splicing factors, proteins responsible for the removal of non-coding intronic sequences and inclusion of coding exons during the processing of a pre-mRNA transcript, were identified in these screens. Decreasing the expression of these splicing factor genes in cultured *Drosophila* cells results in a decrease in the number of lipid droplets formed [5,6] and consistent with these data, our lab has shown that decreasing the expression of members of the U1 and U2 small nuclear ribonucleoprotein (snRNP) complexes in the *Drosophila* fat body results in decreased triglyceride levels [8]. Interestingly, our lab has also identified SR proteins, splicing factors responsible for promoting the use of splice sites and defining intron-exon borders, that regulate triglyceride metabolism [[8], [9], [10]]. Unlike the U1 and U2 snRNP genes, decreasing the expression of the SR protein genes *9G8*, *Tra2*, *RBP1*, and *SF2*, results in an increase in triglyceride storage. The splicing of introns and exons in pre-mRNAs can also be regulated by another group of proteins called heterogenous nuclear ribonucleoproteins (hnRNPs). HnRNPs inhibit splicing by binding closely to SR proteins and masking binding domains for spliceosomes [11]. Our lab has previously identified hnRNPs responsible for the regulation of triglyceride metabolism in the *Drosophila* fat body. Decreasing the expression of the hnRNPs *Hrb98DE*, *Hrb27C*, and *smooth* in the fly fat body results in the accumulation of triglycerides while decreasing fat body *hnRNP-K* and *rump* results in a lean phenotype [12]. However, whether additional hnRNPs play a role in regulating triglyceride metabolism is still unknown.

In this study, we investigated the metabolic functions of the hnRNP *glorund* (*glo*), in the *Drosophila* fat body. Decreasing the expression of *glo* in the fly fat body resulted in a decrease in triglyceride storage throughout the entire fly. However, decreasing fat body *glo* expression increased the amount of triglycerides stored per fat body, but decreased the amount of triglycerides stored in non-fat body tissues. Further analysis indicated a decrease in the expression of the apolipoprotein genes *apolipophorin* (*apolpp*), *microsomal triglyceride transfer protein* (*mtp*), and *apolipoprotein lipid transfer particle* (*apoltp*), suggesting a decrease in lipid transport from the fat body to surrounding tissues. Together, these results identify novel lipid metabolic functions of the *glo* hnRNP and support the link between gene expression and metabolism.

## 2 Materials & methods

### 2.1 Fly genetics

The following fly strains were used in this study: *y* [*1*] sc[*] v [*1*]*; P{y[+t7.7] v[+t1.8] = VALIUM20-EGFP}attP2* (BL#35782, referred to as *UAS-EGFP-RNAi*) and *y* [*1*] sc[*] v [*1*] *sev* [*21*]*; P{y[+t7.7] v[t1.8] = TRiP.HMS00079}attP2* (BL#33668, referred to as *UAS-glo-RNAi*). Flies were grown on standard sugar-cornmeal-yeast food (9 g *Drosophila* agar (Genesee Scientific), 100 mL Karo Lite Corn Syrup, 65 g cornmeal, 40 g sucrose, and 25 g whole yeast in 1.25 L water) at 25 °C on a 12 h:12 h light:dark cycle.

### 2.2 Macromolecule assays

Triglyceride, protein, and DNA were measured in approximately one-week old adult female flies as previously described with minor alterations [13]. Measurements were made from either 2 whole one-week old adult females, 4 cuticles with the remaining fat body attached dissected from one-week old females, or the heads and thoraxes dissected from 4 one-week old females. The wings, legs, and crop remained intact within the dissected head/thorax samples. Whole flies, cuticles with fat bodies, and heads/thoraxes were homogenized in lysis buffer (140 mM NaCl, 50 mM Tris-HCl, pH 7.4, 0.1% Triton-X, and 1X protease inhibitor (Roche)). Proteins were measured using the Pierce BCA Assay kit (Thermo Fisher Scientific) and triglycerides were measured using the Infinity Triglyceride Reagent (Thermo Fisher Scientific) according to manufacturer's instructions. DNA content in fat body dissections was measured using the Quant-iT DNA Assay kit (Invitrogen) according to manufacturer's instructions. Triglyceride levels were normalized by dividing by total protein levels.

### 2.3 Starvation resistance

Groups of approximately ten one-week old adult female flies were placed on starvation media containing 1% agar and the number of flies that were alive was counted every 8 h until all flies were dead. The data was analyzed using the Kaplan-Meier Estimator on the Online Application for Survival Analysis (OASIS; https://sbi.postech.ac.kr/oasis/).

### 2.4 Feeding assay

Feeding was measured in approximately one-week old adult female flies using the CAFÉ assay as previously described [14]. Briefly, flies were placed in an agar vial and given 5% sucrose through a Drummond 5 μl capillary tube (ThermoFisher Scientific). After a 24-h period, the amount of sucrose consumed was measured. The amount of evaporation was accounted for using an agar vial without flies.

### 2.5 RNA isolation, DNase treatment, cDNA synthesis, and quantitative PCR

Fat bodies attached to the cuticle dissected from 15 one-week old adult female flies were homogenized in Ribozol RNA Extraction Reagent (Amresco) according to manufacturer's instructions. 5 μg of extracted RNA samples was DNase treated using the DNA-Free Kit (Ambion) and 0.25 μg of DNased RNA samples was reverse transcribed using qScript XLT cDNA Supermix (QuantaBio) according to manufacturer's instructions. cDNA was combined with 200 or 300 nM of forward and reverse primers, 2x Perfecta SYBR Green (QuantaBio) and water to perform quantitative PCR (qPCR). The following genes were amplified: *glo* (*CG6946*), *rp49* (*CG7939*), *Apoltp* (*CG15828*), *Mtp* (*CG9342)*, *apolpp* (*CG11064*), and *bmm* (*CG5295*). *Glo* and *mtp* primers were used at 300 nM concentrations while the rest were used at 200 nM. The primers for each gene were:

*Glo* forward: 5′ GAACCAATCCGACCAGCTAA 3’

*Glo* reverse: 5′ GCGTCTTTCCGTCGTAGAAC 3’

*Rp49* forward: 5′ GACGCTTCAAGGGACAGTATCTG 3’

*Rp49* reverse: 5′ AAACGCGGTTCTGCATGAG 3’

*Apoltp* forward: 5′ GTTCGAGGTGAGTGGTTGGT 3’

*Apoltp* reverse: 5′ AGCTGCGTCTCATTGGAGAT 3’

*Apolpp* forward: 5′ ATCGGCTCAACACAAAAACC 3’

*Apolpp* reverse: 5′ AGGCAAAAGCGATCTCAAAA 3’

*Mtp* forward: 5′ GTGGGAAGCTTCGTGAAGAG 3’

*Mtp* reverse: 5′ AAAACGCGATACCATTCGAG 3’

*Bmm* forward: 5′ ACGTGATCATCTCGGAGTTTG 3’

*Bmm* reverse: 5′ ATGGTGTTCTCGTCCAGAATG 3’

Quantitative PCR was conducted as follows: 3 min at 95 °C; 40 cycles of: 30 s at 95 °C, 1 min at 60 °C, and 30 s at 72 °C; with a melt curve. Relative quantities were obtained using standard curves and the expression of each gene was normalized to *rp49* expression.

### 2.6 Statistical analysis

A student's t-test was used to compare the experimental *yolk-Gal4* > *gloRNAi* flies to the controls for each assay conducted, except the starvation resistance experiment. Data for the starvation resistance experiment was analyzed using the Kaplan Meier Estimator in OASIS. The Log Rank test was used to determine any difference in mean lifespan of starved flies. The Fisher's Exact test was used to determine differences in 50% mortality. A p-value of <0.05 was considered significant for all statistical analyses.

## 3 Results

### 3.1 glo *regulates triglyceride storage*

**Fig. 1:**
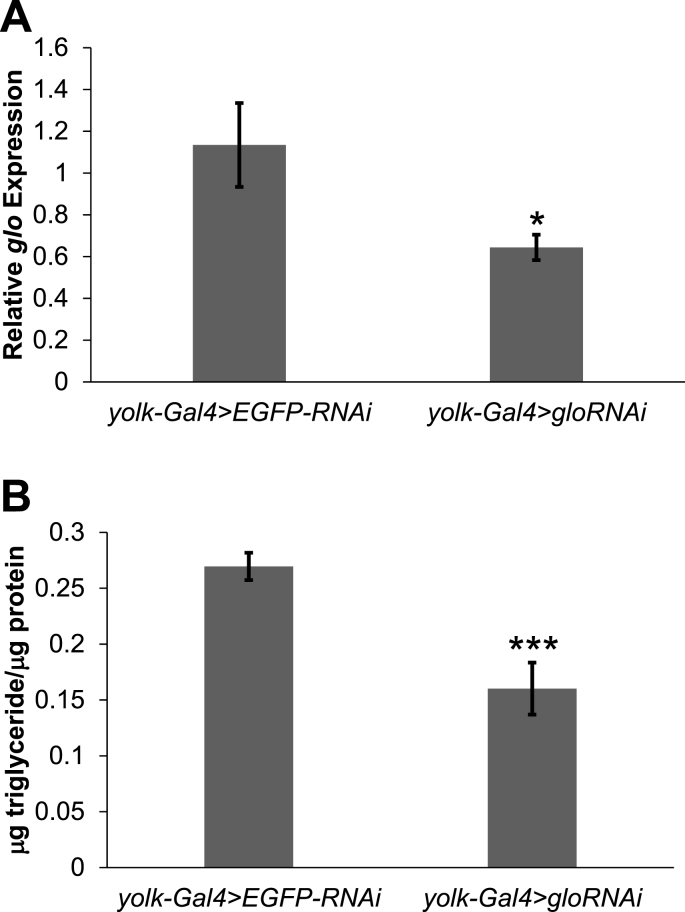
**Decreasing fat body *glorund (glo)* expression decreases triglyceride storage. (A)** RNA was isolated from cuticles with fat body attached dissected from one-week old female *yolk-Gal4* > *EGFP-RNAi* and *yolk-Gal4* > *gloRNAi flies* and *glorund (glo)* expression was measured using qPCR. *glo* expression was normalized to *rp49* expression and average *glo/rp49* ratios are shown ± standard error. **(B)** Triglyceride and protein levels of one-week old adult, female *yolk-Gal4* > *gloRNAi* flies were measured and compared to *yolk-Gal4* > *EGFP-RNAi* controls. The bars represent average triglyceride/protein ratios and error bars represent ±standard error. *p < 0.05; ***p < 0.001 by a student's t-test.

To determine if the heterogeneous ribonucleoprotein glorund (glo) has any metabolic function, we induced RNA interference (RNAi) towards *glo* in the *Drosophila* fat body using the Gal4/UAS system and measured triglyceride levels. Decreasing *glo* expression in the fly fat body (Fig. 1A) resulted in a decrease in triglyceride storage in whole flies as compared to the control (Fig. 1B). This suggests a metabolic role of *glo* in the storage of the major energy storage molecule, triglyceride.

**Fig. 2:**
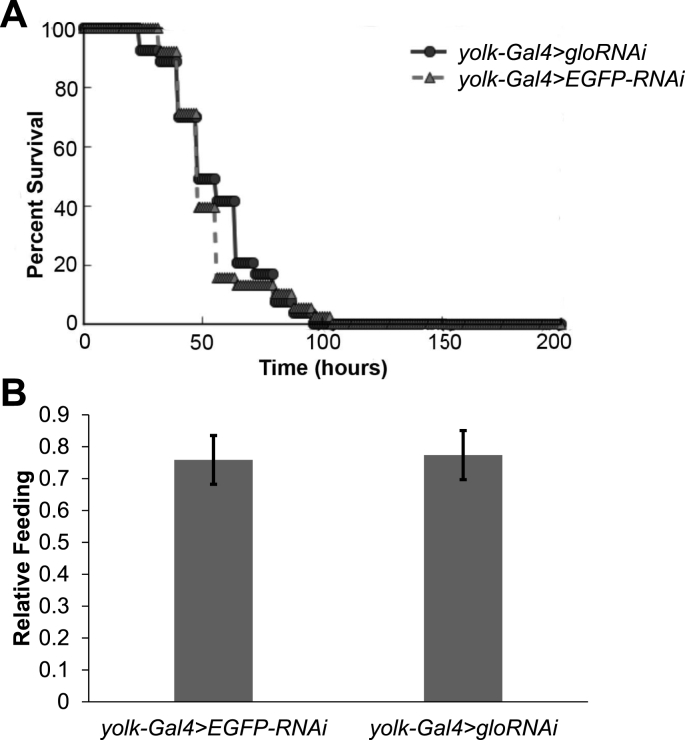
**Decreasing *glo* expression in the *Drosophila* fat body did not affect starvation resistance or feeding. (A)** One-week old, female *yolk-Gal4* > *gloRNAi* and *yolk-Gal4* > *EGFP-RNAi* flies were put on 1% agar starvation media and the number of flies was measured every 8 h. Survival curves were analyzed for mean lifespan using the Kaplan-Meier Estimator on OASIS and p < 0.01 based on Log Rank test with Bonferroni correction. **(B)** Food consumption in adult, female *yolk-Gal4* > *gloRNAi* flies was measured and compared to the *yolk-Gal4* > *EGFP-RNAi* control flies. Feeding was monitored over a 24-h period using the CAFE assay. The bars represent average food consumption ± standard error.

Given the decrease in triglyceride levels seen in *glo*-RNAi flies, we hypothesized that these flies would not survive as well under starvation conditions. To test this hypothesis, we put *glo*-RNAi flies on starvation media (1% agar) and counted the number of flies every 8 h. Surprisingly, the decrease of triglyceride levels in *glo*-RNAi flies had no effect on starvation resistance as compared to the controls (Fig. 2A). This result suggests that despite having less energy storage molecules, *glo*-RNAi animals are able to produce enough energy under starvation conditions to survive similarly to control flies.

### 3.2 Glo *controls the triglycerides stored in the fat body as well as the triglycerides stored in the head and thorax*

It is possible that the decreased triglyceride storage phenotype in *glo*-RNAi flies is due to a decrease in feeding. To test this hypothesis, food consumption over a 24-h period was measured. Feeding was found to be similar in these flies as compared to the control flies (Fig. 2B), indicating that the decrease in triglycerides seen in *glo*-RNAi flies was not due to decreased food consumption.

**Fig. 3:**
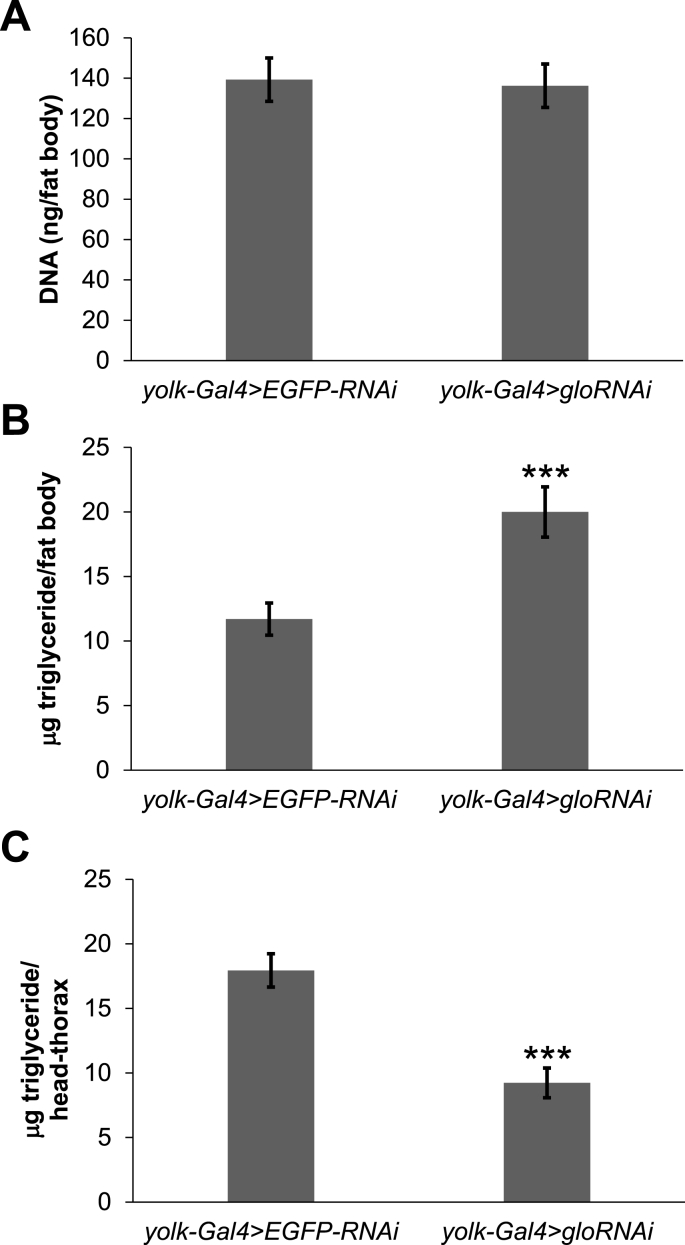
**Decreased expression of *glo* in the *Drosophila* fat body increased the amount of triglycerides stored per fat body and decreased the amount of triglycerides stored in non-fat body tissues.** Abdominal cuticle with fat body attached was dissected from one-week old female *yolk-Gal4* > *EGFP-RNAi* and *yolk-Gal4* > *gloRNAi* flies and **(A)** total DNA content and **(B)** triglyceride/fat body were measured. **(C)** Triglyceride/head-thorax was also measured in combined heads and thoraxes from one-week old adult female *yolk-Gal4* > *EGFP-RNAi* and *yolk-Gal4* > *gloRNAi* flies. Bars represent averages and error bars represent ±standard error. ***p < 0.001 by a student's t-test.

The lean phenotype in *glo*-RNAi flies could also be due to a decrease in the number of fat cells produced, a decrease in the amount of triglyceride stored in these cells, or a combination of both. To test whether the number of fat cells were affected when *glo* was decreased in the fat body, DNA levels were measured in cuticles with fat body attached and used as a surrogate for cell number as previously described [13]. There was no difference in the amount of DNA per fat body in *glo*-RNAi flies compared to the controls (Fig. 3A), suggesting no change in the number of fat body cells. Fat body triglyceride levels were also measured and normalized per fat body to determine if the triglyceride understorage phenotype was due to a decrease in the amount of triglyceride stored in each fat body. Surprisingly, the *glo*-RNAi flies had an increase in triglycerides stored per fat body (Fig. 3B), not the expected decrease. Since triglyceride storage was observed to be less in whole *glo*-RNAi flies, but fat body triglycerides are higher, we hypothesized that triglycerides stored in non-fat body tissues would be less in *glo*-RNAi flies. To test this hypothesis, triglycerides were measured in samples consisting of both heads and thoraxes dissected from *glo*-RNAi flies and these levels were found to be decreased compared to controls as expected (Fig. 3C). Together, these data suggest that *glo* functions in the fat body to limit fat body triglyceride storage and promote the storage of triglycerides in non-fat body tissues.

### 3.3 glo *is important for the expression of the lipid transport genes* apoltp*,* mtp*, and* apolpp

Since fat body triglycerides are elevated in *glo*-RNAi flies, but head/thorax triglycerides are decreased, it is possible that triglycerides are not being transported appropriately among the fat body, intestine and other tissues for storage and *glo* plays a role in this lipid transport function of the fat body. To transport dietary lipids from the intestine to surrounding tissues for usage or storage, lipids are packaged with a major lipoprotein called lipophorin (Lpp) that is expressed in the fat body. The major apoproteins found in Lpp are apolipophorin I and II, encoded by the *apolpp* gene. For proper maturation of Lpp, another protein called microsomal triglyceride transfer protein (mtp) is required. Once the lipid laden Lpp molecule arrives at a target tissue, the lipid transfer particle (LTP), encoded by the *apoltp* gene, is necessary for the interaction of lipophorin with specific tissues and the lipid transfer process. All of these proteins are expressed in the fat body and work to transport and transfer lipids through the hemolymph to tissues throughout the fly [15]; whether *glo* functions in lipid breakdown and transport is not known.

**Fig. 4:**
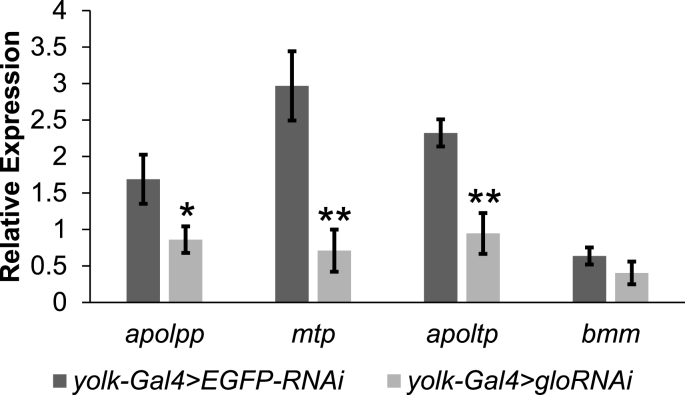
**Decreasing *glo* expression in the *Drosophila* fat body reduced expression of the lipid transport genes *apolpp*, *mtp*, and *apoltp*.** qPCR was used to measure the expression of *apolpp*, *mtp*, *apoltp*, and *brummer* (*bmm*) genes in cuticles with fat body attached dissected from *yolk-Gal4* > *gloRNAi* flies and compared to *yolk-Gal4* > *EGFP-RNAi* controls. Expression of each gene was normalized to *rp49* levels. The bars represent average gene expression and error bars represent ±standard error. *p < 0.05 and **p < 0.01 by a student's t-test.

To further investigate whether *glo* functions in the fly fat body to regulate lipid transport, we examined the effects of decreasing *glo* levels on the expression of genes that regulate lipid transport and breakdown. The relative expression of *apoltp*, *mtp*, *apolpp*, and the triglyceride lipase *brummer* (*bmm*) was determined by performing qPCR. Interestingly, the expression of *apoltp*, *mtp*, and *apolpp* were found to be decreased in *glo*-RNAi flies as compared to the control, while the expression of *bmm* was unchanged (Fig. 4). Together, these data suggest that *glo* is playing a role in the expression and/or stability of these important lipid transport genes or mRNAs to regulate overall triglyceride homeostasis throughout the fly.

## 4 Discussion

In this study, we have characterized the role of the hnRNP glorund (glo) in triglyceride metabolism in the fat body of *Drosophila*. Interestingly, decreasing the expression of *glo* in the fat body resulted in an increase in fat body triglyceride storage but a decrease in triglycerides in non-fat body tissues. One potential explanation for this phenotype is that fatty acid release from triglyceride and then subsequently from the fat body into the hemolymph may be defective. However, if this explanation were true, we would expect that *glo*-RNAi flies would be sensitive to starvation compared to controls and we found that *glo*-RNAi flies had no change in starvation resistance. In addition, we observed that decreasing *glo* levels in the fat body had no effect on the expression of the major adipose triglyceride lipase *brummer* (*bmm*); this together with the starvation resistance data suggests that lipolysis and fatty acid release are occurring normally in *glo*-RNAi flies. Another hypothesis explaining the difference in triglyceride levels in fat body and non-fat body tissues is that *glo* could affect the transport of triglycerides from the fat body to surrounding non-fat body tissues. Consistent with this hypothesis, *glo*-RNAi flies had decreased expression of the lipoprotein genes *apoltp*, *mtp*, and *apolpp*, all of which function to package lipids in the fat body and transport them to non-fat body tissues [15].

The apolipoprotein genes *apolipophorin* (*apolpp*), *apoliprotein lipid transfer particle* (*apoltp*), and *microsomal triglyceride transfer protein* (*mtp*) encode the *Drosophila* homologues of the apoB-containing lipoproteins found in humans [15]. *Apolpp* encodes for the major hemolymph lipid transporter, lipophorin (Lpp), which moves through the fly hemolymph transporting lipids to and from different non-fat body tissues [15]. When Lpp arrives at the fly gut, lipid transfer particles (LTP), encoded by *apoltp*, load Lpp with dietary fats to be transported to non-gut tissues throughout the fly [15]. In addition, microsomal TAG transfer particle (mtp), encoded by *mtp*, is necessary for the maturation of Lpp and LTP in the fat body [15]. Previous studies have found that *mtp*-RNAi flies accumulate immature apolpp precursors in the fat body and are deficient in mature Lpp in the fly hemolymph [15]. Since *mtp* expression was decreased in *glo*-RNAi flies, a decrease in microsomal triglyceride transfer particles could have limited the maturation and secretion of Lpp and LTP from the fat body, thus decreasing the transport of lipids from the gut and fat body to non-fat body tissues. Since the expression of each of the fly apoB-containing lipoproteins (*mtp, apoltp,* and *apolpp*) was decreased, it is also possible that the lack of all three of these genes may be synergistically contributing to the lipid accumulation in the fat body of *glo*-RNAi flies and the decreased lipid storage in the heads/thoraxes of these same flies.

Previous studies have found that by blocking Lpp-dependent lipid import and export in the fat body of *Drosophila*, triglyceride levels are maintained in the fat body and there is a decrease in total triglycerides in wing imaginal discs and the brain [15]. These findings are consistent with our results that *glo*-RNAi flies, which have decreased expression of *apolpp*, also have a decrease in triglyceride levels in the head and thorax. However, in previous studies where apolipoprotein lipid transfer particles are decreased using RNAi, a decrease in lipid storage was observed [15]. While this phenotype is the opposite of what we see in *glo*-RNAi flies that have decreased *apoltp* expression, this inconsistency could be due to the fact that the previous study mentioned above was performed in *Drosophila* larvae while our experiments were performed in adult flies, stages of development that have very different metabolic needs.

Glorund is well known to function as an RNA binding protein (RBP) during *Drosophila* development, specifically by interacting with a UA-rich motif found in the 3’ UTR of the developmental gene *nanos* [16,17]. The interaction of glo with the *nanos* mRNA prevents the localization and translation of *nanos* in the anterior pole and aids in establishing the anterior-posterior gradient of developing embryos [16]. In addition, glo has been shown to be a translational repressor of *nuclear-encoded respiratory chain complex* (*nRCC*) mRNAs to help maintain mitochondrial function [18]. These studies show that glo not only plays a role in development, but also has metabolic functions, through the binding and localization of specific mRNAs. It is possible that glo binds to the mRNAs made from lipid transport genes like *apolpp*, *apoltp* and *mtp* to regulate their expression or stability as the levels of these mRNAs are decreased in *glo*-RNAi flies. It is also possible that glo could regulate the splicing of these lipid transport genes along with their expression, (specifically the splicing of *apolpp*, since *mtp* and *apoltp* only have one isoform) and further experimentation will be necessary to differentiate among these possibilities.

Our lab has previously identified a number of hnRNPs whose fat body expression is necessary for normal lipid storage; one example is *Hrb27C*, an hnRNP whose knockdown results in excess triglyceride storage [12]. When we further examined the triglyceride phenotype of fat body-specific *Hrb27C*-RNAi flies, we observed that the amount of triglyceride stored in each fat body cell was also increased in these flies, and this lipid accumulation phenotype could partially be due to blunted lipid breakdown [12]. Interestingly, Hrb27C forms a complex with glo to regulate localization of *gurken* mRNAs and dorsal-ventral polarity in the *Drosophila* embryo [19]. Therefore, it is possible that glo could be acting with Hrb27C to regulate lipid metabolism in the fly fat body. Consistent with this hypothesis, both glo and Hrb27C proteins have been found to localize on the surface of lipid droplets [20]. However, decreasing the expression of *glo* in the fly fat body resulted in decreased overall triglyceride storage, possibly due to a defect in lipid transport, which is a different phenotype than what we observed in *Hrb27C*-RNAi flies [12], perhaps indicating that glo and Hrb27C may act independently to regulate fat body lipid metabolism. Therefore, it is possible that different hnRNPs have different mechanisms whereby they regulate lipid storage and further experiments aimed at characterizing how different hnRNPs regulate triglyceride levels will be necessary to better understand the metabolic functions of this class of proteins.

In summary, we describe here novel metabolic functions for the hnRNP, glorund, in the *Drosophila* fat body. We hypothesize that glo targets specific mRNAs to regulate the transport of lipids from the gut and fat body to the rest of the fly. *Glorund* is highly conserved and is the *Drosophila* homolog of hnRNP F/H family members in humans [21]. Due to the conservation of *glorund*, a better understanding of the metabolic functions of this hnRNP could help further our understanding of the genes and mechanisms regulating lipid metabolism and transport in humans and help with the development of additional treatments and preventatives for metabolic diseases such as obesity.

## CRediT authorship contribution statement

**Annabella M. Kolasa:** Investigation, Formal analysis, Writing - original draft, Visualization. **Jasleen K. Bhogal:** Investigation, Formal analysis, Writing - review & editing. **Justin R. DiAngelo:** Conceptualization, Methodology, Investigation, Formal analysis, Resources, Writing - review & editing, Visualization, Supervision, Project administration, Funding acquisition.
